# Differences in healthcare costs in youths with conduct disorders in rural vs. urban regions: an analysis of German health insurance data

**DOI:** 10.1186/s12913-018-3520-0

**Published:** 2018-09-14

**Authors:** Heike Gerhardt, Monika Heinzel-Gutenbrunner, Christian J. Bachmann

**Affiliations:** 1 Department of Child and Adolescent Psychiatry, Philipps-University Marburg, Hans-Sachs-Str. 4-6, DE-35039 Marburg, Germany; 2 Department of Child and Adolescent Psychiatry, LVR-Klinikum Düsseldorf/ Kliniken der Heinrich-Heine-Universität Düsseldorf, Bergische Landstraße 2, DE-40629 Düsseldorf, Germany

**Keywords:** Adolescents, Children, Conduct disorder, Cost analysis, Germany, Health insurance company, Rural, Secondary data, Health services utilisation, Urban

## Abstract

**Background:**

For children and adolescents with mental health problems, there is a lack of data as to whether the type of residential area (urban vs. rural) influences healthcare costs for affected individuals. The aim of this study was therefore to explore potential urban vs. rural healthcare cost differences in children and adolescents with conduct disorder (CD), one of the most frequent and cost-intensive child and adolescent psychiatric disorders. Additionally, we aimed to compare healthcare costs of youths with CD, and of youths without this diagnosis.

**Methods:**

We analysed data from a German health insurance company, extracting all youths with a CD diagnosis in 2011 (CD group; *N* = 6337), and an age- and sex-matched group without this diagnosis (control group). For both groups, annual costs per person for outpatient and inpatient healthcare were aggregated, stratified by area of residence (urban vs. rural).

**Results:**

While mean annual overall costs in the CD group did not differ significantly between urban and rural areas of residence (2785 EUR vs. 3557 EUR, *p* = 0.253), inpatient treatment costs were significantly higher in rural areas (2166 EUR (60.9% of overall costs) vs. 1199 EUR (43.1% of overall costs), *p* < 0.0005). For outpatient healthcare costs, the reverse effect was found, with significantly higher costs in individuals from urban areas of residence (901 EUR (32.3% of overall costs) vs. 581 EUR (16.3% of overall costs), *p* < 0.0005).

In the control group, no significant rural vs. urban difference was found for either overall health costs, inpatient or outpatient costs. Mean overall costs in the CD group were four times higher than in the control group (3162 (±5934) EUR vs. 795 (±4425) EUR).

**Conclusions:**

This study is the first to demonstrate urban vs. rural differences in healthcare costs among youths with CD. The higher costs of inpatient treatment in rural regions may indicate a need for alternative forms of service provision and delivery in rural settings.

## Background

According to the International Classification of Diseases, 10th revision (ICD-10 [1]), conduct disorder and oppositional defiant disorders (within the following text summarised under the term “conduct disorders”) can be defined as “a repetitive and persistent pattern of dissocial, aggressive or defiant conduct” that “violates the basic rights of others or age-appropriate societal expectations”. Conduct disorders are among the most common disorders in child and adolescent psychiatry, with a prevalence of approximately 5–7% [2, 3]. In daily practice, children with conduct disorders constitute a significant portion of outpatients, e.g. 30% of a typical general practitioner’s (GP) child consultations, and 45% of community child health referrals in the United Kingdom (UK) [4].

Psychiatric comorbidity in conduct disorders is common, and includes amongst others attention-deficit/hyperactivity disorder (ADHD), depression, anxiety disorders, and substance misuse [5]. Without treatment, in up to 50% of individuals symptoms persist into adulthood, leading to antisocial personality disorder, criminal offending, or incarceration [6, 7].

Studies from the UK and from Germany demonstrate an above-average healthcare utilisation and subsequent costs in individuals with conduct disorders [8–11].

In a British follow-up study, individuals diagnosed with conduct disorder in childhood had caused 8.8 times higher direct health costs in adulthood than same-aged individuals without childhood conduct disorder [10]. The same study provided estimates of lifetime costs for children with conduct disorder, which can amount to ≥ £1 million. In a German study, healthcare expenses for adolescents with conduct disorders were 3.8 times higher than in youth without a conduct disorder diagnosis [9].

Apart from health services utilisation, conduct disorder related costs are also incurred in the education system, within the family (through lost productivity), and – due to increased delinquency – in the justice system [11, 12]. Elevated levels of conduct problems in childhood are particularly associated with increased justice costs in adulthood [13]. A study of economic consequences of adolescent antisocial behaviour from the United States of America (USA) estimated additional costs at age 24 of 2.1–3.7 million USD for a typical criminal career [14]. Additionally, studies have demonstrated that even comorbid conduct disorder in children and adolescents with depression or ADHD doubled annual service use costs [15, 16].

While there is evidence that in adults with psychiatric disorders the type of residential area (urban vs. rural) significantly influences health service use and associated healthcare costs [17, 18]), such data is not available for children and adolescents.

The few existing studies on urbanicity in relation to conduct disorders mainly focus on the influence of urban or rural environments on the prevalence of conduct disorders and antisocial behaviour: A study in US schoolchildren showed that while behaviour problems at home did not vary significantly across urban and rural settings, children in urban settings exhibited higher rates of school conduct problems [19]**.** Harden et al. studied 10- to 17-year olds in the UK, and did not find support for the hypothesis that living in densely populated counties influenced youth delinquency [20].

There is evidence that children and adolescents with conduct disorder frequently show complex and multiple service use patterns, e.g. primary healthcare, specialist child mental health services, emergency department visits, behavioural therapy, social services, contacts with the criminal justice system etc. [11, 21]. In rural areas, such a comprehensive array of services may not be as widely available as in urban areas [22–24]. In addition to the availability of services, factors like social visibility in small communities or difficulties associated with travelling to consult a mental health professional might also influence service use and associated healthcare costs in rural areas [25, 26].

This study therefore aimed to explore the following questions:

1. Are there differences in overall, outpatient and inpatient healthcare costs in children and adolescents with a diagnosis of conduct disorder from urban vs. rural places of residence?
2. Are there differences in the composition of healthcare costs in relation to type of cost and type of consulted medical specialty?
3. Are there differences in overall, outpatient and inpatient healthcare costs in children and adolescents with a diagnosis of conduct disorder and of youths without this diagnosis?

## Methods

### Data and sample

This study used secondary data from the AOK Nordost health insurance company, which is a statutory health insurance company that covers about 1.8 million inhabitants of the German federal states Berlin, Mecklenburg-Western Pomerania and Brandenburg. Nearly 90% of children and adolescent in Germany are insured in a statutory health insurance company, whereas the remainder are privately insured [27]. Although there are several differences between the statutory and private system, both provide full-coverage health insurance. Insurees from a statutory health insurance company can go to almost any doctor or hospital in Germany in order to receive medical care, and have it reimbursed by their insurance company.

As compared with the entire German population, AOK insurees have a slightly lower socio-economic status, and more psychiatric morbidity [27].

Out of all children and adolescents who were insured in each quarter of 2011, we extracted all who were 5 to 18 years old in 2011, and for whom one of the following ICD-10 conduct disorder diagnoses had been documented: F90.1, F91.0, F91.1, F91.2, F91.3, F 91.8, F91.9, F92.0, F92.8 or F92.9. These children constituted the conduct disorder group. For the control group, a random sample of age-, sex-, and postcode-matched children without any of the afore-mentioned conduct disorder diagnoses in the years 2006–2011 was extracted from all AOK Nordost insurees in 2011.

For both groups, the prevalence of comorbid psychiatric diagnoses (remaining ICD-10 F codes) was evaluated.

Annual healthcare costs per individual for both groups were calculated by adding up the following cost data from the AOK data for the calendar year 2011: inpatient hospital costs, outpatient GP costs, outpatient specialist costs, prescription of pharmaceuticals, rehabilitation costs, costs for medical aids, and medical remedies, and reimbursement of healthcare-related travel expenses.

The variable “urban” vs. “rural” region was operationalised conferring to the number of inhabitants (urban: ≥100,000 inhabitants, rural: < 100,000 inhabitants), according to the definition of the German Federal Institute for Research on Building, Urban Affairs and Spatial Development (Bundesinstitut für Bau-, Stadt- und Raumforschung) [28].

### Statistical methods

Because of the non-normal distribution of the data, Mann-Whitney tests were computed for the analysis of differences between health services utilisation costs in rural and urban regions. Additional dependent-samples analyses of variance were applied, based on the matched index-control pairs. This model enables the analysis of interactions between the conduct disorder group and the control group, and between urban and rural regions. In addition to *p*-values, effect sizes were calculated, based on the test statistics of the Mann-Whitney test.

## Results

### Sample characteristics

The conduct disorder group and the control group each consisted of 6337 individuals, 68.8% of which were male. The mean age was 11.2 (±3.7, range: 5–18) years. In both groups, 43.2% of insurees were from the federal state of Berlin, 30.6% were from Brandenburg, and 26.1% were from Mecklenburg-Western Pomerania. 51.2% were from an urban area of residence, and 48.8% lived in a rural area of residence. The proportion of individuals of non-German nationality was 26.2% in Berlin, 3.1% in Brandenburg, and 2.3% in Mecklenburg-Western Pomerania.

In the conduct disorder group, 93.3% of urban residents, and 92.7% of rural residents had at least one psychiatric comorbidity (*p* = 0.348). In the control group, the corresponding prevalence rates were 24.0%, and 25.8%, respectively (*p* = 0.086).

### Cost analysis

#### Conduct disorder group

The mean overall annual healthcare costs per person were 3162 (±5934) EUR, with overall healthcare costs not differing significantly between patients from urban and from rural regions (2785 EUR vs. 3557 EUR, *p* = 0.253).

The detailed composition of costs by type of healthcare cost and area of residence (rural vs. urban) is shown in Table 1. Both in rural and urban areas, inpatient costs constituted the largest part of overall costs, followed by outpatient costs, and expenses for pharmacotherapy. Inpatient healthcare costs in rural regions of residence were significantly higher than those in urban areas, while outpatient healthcare costs were significantly higher in urban regions of residence.

**Table 1:** Mean annual costs (in EUR) in 2011, by group, cost type and area of residence

Conduct Disorder Group							Control Group					
Cost type	Urban	% of overall costs	Rural	% of overall costs	P-value	Effect size (d)	Urban	% of overall costs	Rural	% of overall costs	P-value	Effect size (d)
Inpatient services	1198.90 (±4564.79)	43.1	2166.18 (±5911.83)	60.9	< 0.0005	0.18	180.54 (±1504.75)	23.3	290.33 (±1714.29)	35.5	0.007	0.07
Outpatient services	900.81 (±999.41)	32.3	581.10 (±624.28)	16.3	< 0.0005	0.38	229.51 (±406.60)	29.7	210.82 (±259.31)	25.8	0.030	−0.05
Pharmacotherapy	209.45 (±802.90)	7.5	272.14 (±2189.64)	7.6	0.132	−0.02	179.83 (±5061.23)	23.2	114.73 (±699.86)	14.0	0.471	0.02
Additional therapies	200.08 (±438.02)	7.2	174.97 (±542.15)	4.9	0.042	−0.05	82.76 (±382.29)	10.7	69.10 (±235.91)	8.5	0.089	−0.04
Social paediatric centre	171.37 (±451.56)	6.2	161.10 (±363.51)	4.5	< 0.002	0.07	42.16 (±325.12)	5.4	29.83 (±168.55)	3.7	0.056	−0.05
Transportation costs	66.10 (±314.32)	2.4	119.88 (±488.39)	3.4	< 0.0005	0.13	13.73 (±81.33)	1.8	28.35 (±190.25)	3.5	< 0.0005	0.10
Medical aids and applicances	31.22 (±373.67)	1.1	53.25 (±513.27)	1.5	0.050	0.05	39.89 (±488.72)	5.2	49.68 (±490.56)	6.1	0.427	0.02
Rehabilitation	3.79 (±158.83)	0.1	14.44 (±366.27)	0.4	0.130	0.04	6.61 (±224.36)	0.9	13.71 (±327.58)	1.7	0.312	0.03
Direct payments	4.84 (±44.08)	0.2	10.42v(±72.85)	0.3	< 0.0005	0.09	2.76 (±33.79)	0.4	8.43 (±58.31)	1.0	< 0.0005	0.12
Overall Costs	**2784.60 (±5037.73)**		**3557.41 (±6723.80)**		**0.253**	**0.03**	**773.70 (±5650.52)**		**816.90 (±2578.49)**		**0.698**	**0.01**

Data on outpatient healthcare costs by type of physician (general practitioners and selected specialties) are presented in Table 2. The highest outpatient costs were incurred by contacts with specialist doctors, i.e. child and adolescent psychiatrists, and paediatricians. Costs per specialty were significantly higher in insurees from urban areas of residence than in those from rural areas, while costs for GP consultations were significantly higher in rural areas.

**Table 2:** Mean annual costs (in EUR) in 2011 for outpatient health services, by group and specialty

	Conduct Disorder Group				Control Group			
Specialty	Urban	Rural	*P*-value	Effect size (d)	Urban	Rural	*P*-value	Effect size (d)
Child and adolescent psychiatrist/ psychotherapist	493.92 (±899.50)	271.80 (±523.67)	< 0.0005	0.23	31.28 (±235.10)	20.56 (±155.65)	0.033	−0.05
Paediatrician	165.92 (±233.14)	95.64 (±135.60)	< 0.005	0.24	83.85 (±114.64)	69.61 (±92,96)	< 0.0005	−0.14
General practitioner	48.55 (±87.57)	57.83 (±79.86)	< 0.0005	0.15	33.90 (±59.09)	43.71 (±65.74)	< 0.0005	0.16

#### Control group

In the control group, overall healthcare costs per person were 795 (±4425) EUR. Overall costs did not show a significant difference between urban and rural areas of residence (774 EUR vs. 817 EUR, *p* = 0.698) (Table 1).

In rural areas, inpatient costs constituted the largest part of overall costs (35.5%), while in urban areas costs for outpatient healthcare were the most important cost type (29.7%). Costs for inpatient treatment in rural regions of residence were significantly higher than those in urban areas. For outpatient healthcare costs, this relationship was inverse.

Visits to paediatricians incurred the largest part of outpatient costs (Table 2). In urban areas, costs for specialist doctor consultations were significantly higher than in rural areas, and in rural areas, GP costs were significantly higher than in urban areas of residence.

In comparison, the mean annual overall healthcare costs per person in the conduct disorder group were 3.98-fold higher than those in the control group. With regard to inpatient healthcare, costs in the conduct disorder group were 6.64-fold (urban) and 7.46-fold higher (rural), respectively, than in the control group.

## Discussion

The main finding of this exploratory study is the inverse relationship between inpatient and outpatient costs in the conduct disorder group: While inpatient costs were significantly higher in individuals from rural areas, outpatient costs were significantly higher in individuals from urban areas of residence.

A possible cause for these cost differences is an unequal availability of medical resources in urban vs. rural areas of residence. In Germany, there are considerable differences between rural and urban areas of residence in regard to the number and density of statutory health insurance-accredited physicians and mental health professionals. In 2011, for example, the most densely populated German federal state of Berlin (about 3950 inhabitants/km^2^) had the highest density of registered psychotherapists of all federal states (60.3 per 100,000 inhabitants). In contrast, the two least populated, rural federal states Brandenburg and Mecklenburg-Western Pomerania (about 70–80 inhabitants/km^2^) had a ratio of 12.5 and 10.9 psychotherapists per 100,000 inhabitants, respectively [29]. This urban vs. rural difference also applies to child and adolescent psychiatrist density: With the national average index set at 1, the index for Berlin is 2.0, while for Brandenburg and Mecklenburg-Western Pomerania indices are < 1, i.e. below the national average [30].

The above-mentioned differences in the availability of mental health professionals might have influenced service utilisation in the following way: In rural regions with a scarcity of office-based child and adolescent psychiatrists, cases where child and adolescent psychiatric diagnostic or therapeutic expertise is indispensable, are directly referred into hospital for further work-up, thus causing much higher inpatient treatment costs. The high costs for inpatient treatment (which constitute the largest part of conduct disorder treatment costs) are especially remarkable as there is no good evidence for lasting effects of inpatient treatment for children and adolescents with conduct disorders [2]. Vice versa, in urban areas of residence with a generally good availability of office-based child and adolescent psychiatrists, utilisation of these outpatient services is much higher than in rural regions, resulting in higher total outpatient costs, and – as timely intervention in an outpatient setting can often prevent inpatient admission – in lower inpatient costs. Higher utilisation of specialist physicians by insurees from urban areas in the presence of mental illness, together with higher associated healthcare costs, was confirmed in a recent study from Germany [31].

Yet, an Australian study focusing on mental health services use for anxiety and mood disorders in the general population did not find rural residence itself to be a limiting factor for access to mental health services. Instead, other factors, including education and distress, were more important predictors of mental health services utilisation [32].

Another possible explanation for the differences in inpatient and outpatient healthcare costs found in this study could be an urban vs. rural difference in terms of symptom severity or psychiatric comorbidity. Regarding symptom severity, this parameter was not included in the secondary data underlying this study, thus precluding such an analysis. Concerning psychiatric comorbidity, there were no significant urban vs. rural differences in the respective prevalence rates, which makes an influence of comorbidity on cost differences unlikely.

Concerning the distribution of costs according to service type, our results correspond with an economic analysis of adolescents with conduct disorder by Ewest et al. [9], with inpatient treatment costs being higher than outpatient treatment costs, and these being followed by costs for medication. Our findings in terms of cost distribution also comply with those of Kohlboeck et al. [33], who investigated child behavioural problems in Germany. However, their evaluation of behavioural problems was not based on any ICD-10 diagnosis, but rather on information provided by parents and their evaluation of their child’s utilisation of healthcare services (outpatient and inpatient treatment). In their study, the severity of reported behavioural problems (abnormal vs. normal) correlated with about five-fold higher direct total costs for medical care, with hospital costs surmounting costs for outpatient physician visits.

Regarding the composition of outpatient costs, consultations of child and adolescent psychiatrist consultations naturally yielded the highest costs in the conduct disorder group. Regarding the higher costs for GP consultations in rural areas, these results are in agreement with a recent study demonstrating that children and young people in rural German areas use outpatient general medical care to a greater extent than those in urban areas [34], but also with studies of service use for mental health problems in rural areas in general [35].

In terms of the four times higher overall healthcare costs for children and adolescents with conduct disorders, our data is in line with the study by Ewest et al. [9], who found 3.8 times higher total annual costs for health services utilisation in the conduct disorder group compared to a control group. The even higher ratio in inpatient healthcare costs supports the notion of conduct disorder as a disorder that is difficult to treat within an outpatient setting [36].

Beyond the above-mentioned studies, there are currently no other comparable studies available which focus on urban vs. rural differences in the utilisation of healthcare services and health insurance expenses in youth with psychiatric disorders. Interestingly, our results largely comply with those of the few studies performed on rural vs. urban cost differences in adult psychiatric patients. These studies also did not find rural vs. urban differences in overall healthcare costs [18], and reported lower psychiatric outpatient service use in rural areas [17, 37], and higher inpatient costs in rural areas of residence [18].

While Tiainen et al. [37] and Ziller et al. [17] described higher utilisation of pharmacotherapy in rural areas, this finding could not be replicated in our study. The reason for this discrepancy is probably the fact that according to the guidelines for the management of conduct disorders in children and adolescents [4, 38], pharmacotherapy is not a recommended first-line treatment.

Drawing on our data, an improved healthcare provision for conduct disordered children and adolescents appears to be necessary primarily in rural areas. Given the scarcity of mental health professionals in these areas, improvements in healthcare provision for conduct-disordered youths should focus on dissemination and implementation of evidence-based prevention and intervention programmes [4] that do not draw on medical workforce capacity. A number of parenting programmes meet these criteria, e.g. the 12-week *Incredible Years* programme or the 8-week *Triple P* programme, which do not only improve conduct disorder symptoms, but have also proven to be cost-effective [39–42]. Another well-evaluated intervention is the home-based *Multisystemic Therapy* [43], which has demonstrated superiority over inpatient treatment, both clinically and in terms of cost-effectiveness, [44], and can be run by social workers.

An alternative would be the provision of distance- or internet-based interventions, for whose effectiveness there is a growing body of evidence [45, 46].

Beyond the reduction of healthcare costs, an improved provision of healthcare as delineated above would also have the potential for reducing non-medical costs associated with conduct disorders (e.g. youth welfare measures, special needs schooling, etc.) [13, 42, 47, 48], and, last but not least, reducing the higher mortality in rural areas.

### Strengths and limitations

In terms of limitations, it is important to note that we used secondary data, so no external validation of the conduct disorder diagnoses was possible. Also, the data did not contain information on clinical factors potentially influencing service utilisation and associated costs, e.g. symptom severity [10, 13]). Moreover, the underlying data stems from a single statutory health insurance fund, whose insurees have a slightly lower-than-average socioeconomic status, and an elevated level of mental health problems [27]. Thus, our sample is not representative for the whole of the German population, and may have incurred higher healthcare costs than insurees of other health insurance companies. Information on important confounders, e.g. education or family composition, was not available in the underlying secondary data set [49]. Also, our data does not include information regarding the utilisation of non-medical mental health care, e.g. parent training provided by social services.

In order to match control group and index group, not only age and sex but also the residential area (postcode) was taken into account, in order to serve as a proxy for socioeconomic status. Nevertheless, the lack of individual socioeconomic status data is a significant limitation, which should be mended in future studies, as some studies have demonstrated associations between socioeconomic status and health services use in children, albeit in different directions [50, 51]. Moreover, the utilisation of healthcare services can also be influenced by nationality and ethnicity [52]. Unfortunately, the nationality data provided by the AOK Nordost does not always reflect the original ethnicity, thus precluding further analyses.

Another methodological issue is the definition of areas as urban and rural, with towns with more than 100,000 inhabitants being defined as urban. It is debatable whether this classification is appropriate in the case of Berlin, which simultaneously constitutes a city and a federal state. However, the afore-mentioned classification has also been employed within the German KiGGS study, a representative epidemiological study on child health [53], so we adhered to it for the sake of general comparability.

Additionally, it has to be noted that in Germany the unit costs for inpatient and outpatient treatment do not reflect the real costs of the service providers, but are rather a price negotiated between the health insurance company and the service providers.

Finally, it should be kept in mind that the effect sizes corresponding with the respective significant differences from our analysis are all small in dimension, thus qualifying the urban vs. rural differences.

A strength of this study is its use of administrative data, which allows to study real-world utilization patterns in a large and unselected population without nonresponse or recall bias problems. Moreover, this study is of special interest, as it constitutes the first approach to investigate rural vs. urban differences in the provision of healthcare for children and adolescents with conduct disorders.

## Conclusions

This study is the first to demonstrate urban vs. rural differences in healthcare utilisation and costs among youths with conduct disorder. The higher costs of inpatient treatment in rural regions may indicate a need for alternative forms of service provision and delivery in rural settings.

## Abbreviations

ADHD: Attention-deficit/hyperactivity disorder
CD: Conduct disorder
GP: General practitioner
ICD-10: International Classification of Diseases, 10th revision
UK: United Kingdom
US/USA: United States of America
